# A case of double common bile duct in a deceased donor for transplantation

**DOI:** 10.1007/s00276-017-1874-3

**Published:** 2017-05-18

**Authors:** Hajime Imamura, Susumu Eguchi, A. M. James Shapiro, Tatsuya Kin

**Affiliations:** 1 Department of Surgery, Nagasaki University Graduate School of Biomedical Sciences, Nagasaki, Japan; 2 Clinical Islet Transplantation Program, University of Alberta, Edmonton, AB Canada; 3 Clinical Islet Laboratory, University of Alberta, 210 College Plaza, 8215-112th St, Edmonton, AB T6G 2C8 Canada

**Keywords:** Double common bile duct, Extrahepatic bile duct duplication, Duplication of the common bile duct, Accessory bile duct, Double choledochus

## Abstract

A double common bile duct is extremely rare among the anatomical variations in the biliary tract system. We report an incidentally encountered case of the double common bile duct and discuss the novel anatomical findings of the accessory common bile duct from the viewpoint of embryology. A unique point of our case is that the accessory common bile duct bifurcated at the level of the intrapancreatic bile duct. There is no similar case in the previous literature among type II double common bile duct in the viewpoint of anatomical findings of the accessory common bile duct. We assume that this asymptomatic anatomical variation may be present more commonly, but not diagnosed.

## Introduction

A double common bile duct (CBD) is extremely rare among the anatomical variations in the biliary tract system. Herein, we report a case of double CBD with accessory CBD bifurcated at the level of the intrapancreatic bile duct.

## Case report

**Fig. 1:**
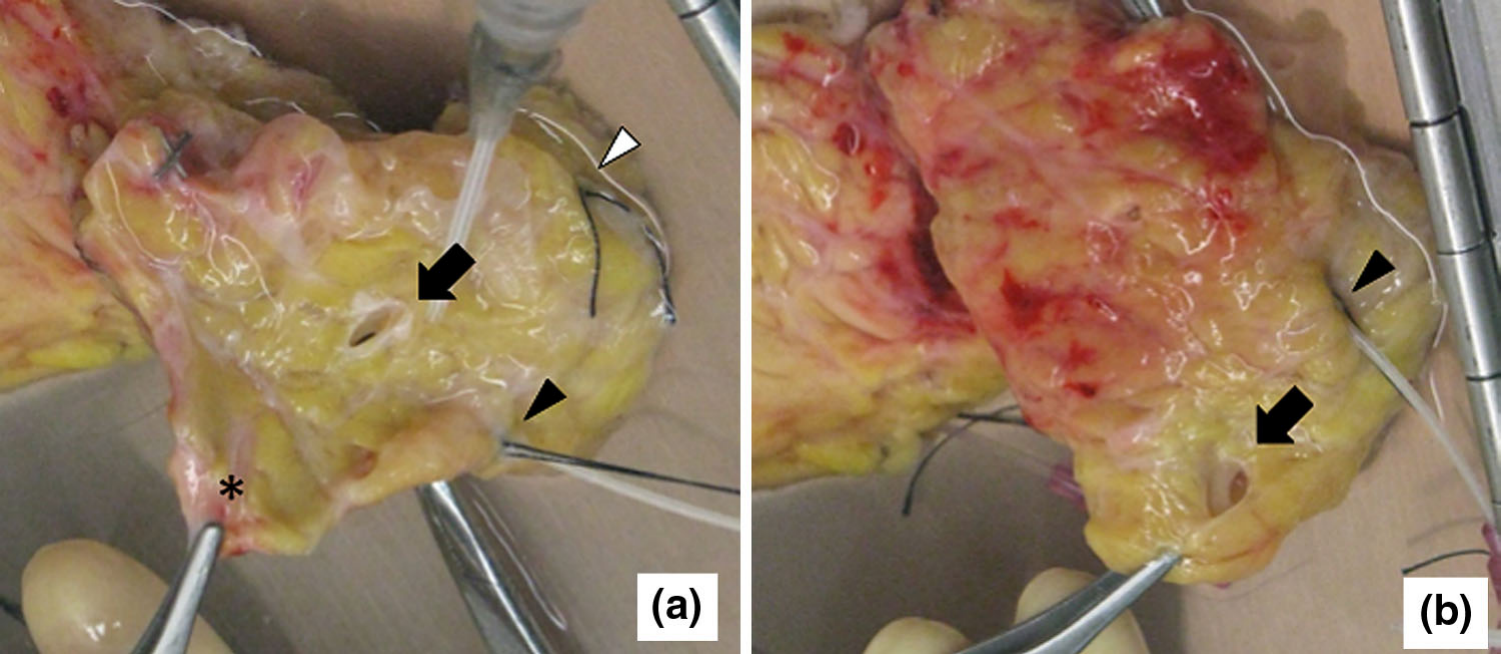
The posterior aspect of pancreas head is shown. A catheter was inserted into the Wirsung duct (*black arrowhead*) for intraductal injection of collagenase solution. The Santorini duct was found to be patent and ligated with *black* silk (*white arrowhead*). **a** The proximal side of the accessory CBD (*black arrow*) was detected at the *middle part* of the groove where the intrapancreatic bile duct was located. The pancreatic lingula (*asterisk*) is retracted by forceps. **b** The distal side of the accessory CBD opened at the inferior and posterior side of the pancreatic head at a distance about 2 cm from the Wirsung duct (*black arrow*)

**Fig. 2:**
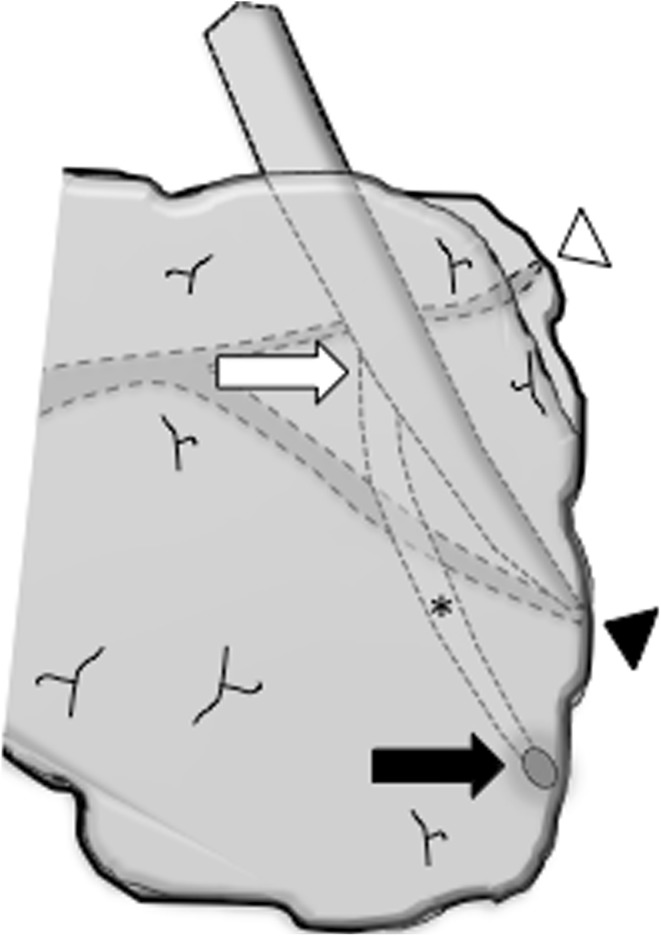
Schema showing the location of the accessory CBD. The bifurcation of the accessory CBD (*white arrow*); The distal end of the accessory CBD (*black arrow*); The accessory CBD inside the pancreatic parenchyma (*asterisk*); The Santorini duct (*white arrow head*); The confluence of the bile duct and the Wirsung duct (*black arrow head*)

A 66-year-old Hispanic female was admitted for cerebrovascular accident. Her past medical history was unremarkable except for splenectomy and cholecystectomy for hemolytic anemia at the age of 35. Her family history was also unremarkable. Laboratory tests revealed normal liver function, serum amylase/lipase levels, and hemoglobin A_1c_. After the diagnosis of brain death was made and a legal consent was obtained from her family, she became a multiple-organ donor. Her pancreas was procured en bloc with the duodenum for islet isolation and transplantation. The islet isolation procedure began with removal of the duodenum and intrapancreatic bile duct from the pancreas. When the intrapancreatic bile duct was dissected from the pancreas, the proximal side of the accessory CBD was detected at the middle part of the groove, where the original CBD was located (Fig. 1a). The accessory CBD was completely embedded inside the pancreatic parenchyma. It ran toward the inferior and posterior side of the pancreatic head. The distal end of the accessory CBD opened into the second portion of the duodenum at a distance about 2 cm from the orifice of Wirsung duct (Fig. 1b). From these findings, we diagnosed this anatomical variation as double CBD with accessory CBD bifurcated at the level of the intrapancreatic bile duct (Fig. 2). We did not detect any anatomical variation in the pancreas, such as pancreas divisum or pancreaticobiliary maljunction.

## Discussion

Many variations have been described and classified in bile duct anatomy [2, 5, 7]. In regards to double CBD, Choi et al. [3] proposed modification of the classification consisting of five-configuration type: (I) CBD with a septum within the lumen; (II) CBD which bifurcates with two independent drainages; (III) double biliary drainage without communicating channels (IIIa, without intrahepatic communicating channels; IIIb, with intrahepatic communicating channels); (IV) double biliary drainage with one or more communicating channels; and (V) a single biliary drainage (Va, without any communication between two extrahepatic bile ducts; Vb, with one or more communication channels). According to this classification, our case is classified as type II. A limited number of reports are found about type II double CBD cases. A unique point of our case is that the accessory CBD bifurcated at the level of the intrapancreatic bile duct. There is no similar case in the previous literature among type II double CBD in the viewpoint of anatomical findings of the accessory CBD. We believe this report has the novelty and value in point of indicating the images of the unprecedented bifurcated pattern of the accessory CBD. This anatomical variation did not interfere with pancreatic enzymatic distension and the subsequent islet isolation process.

The pancreas derives from the dorsal and ventral primordium. During the process of embryonic development, due to the dominant expansion of growth on the left side of the primitive duodenum, the ventral pancreas with the primitive bile duct passively moves to the right and rotates posteriorly until it comes to lie to the left of the duodenum, subsequently fusing to the dorsal pancreas [6]. As a result, the intrapancreatic bile duct runs between the dorsal and ventral pancreas, neither inside the dorsal pancreas nor the ventral pancreas. This configuration is confirmed in human fetal pancreas as well [9]. Also, the intrapancreatic bile duct is covered by the pancreatic lingula [8], which derives from the ventral pancreas. In our case, the proximal side of the accessory CBD was detected at the middle part of the intrapancreatic bile duct when the intrapancreatic duct was removed after the pancreatic lingula was retracted. From these viewpoints, although the accessory CBD was completely embedded inside the pancreas, it is reasonable to assume that the accessory CBD runs in the boundary between the dorsal and ventral pancreas parenchyma. Removal of the intrapancreatic bile duct is our standard procedure during the pancreas preparation for islet isolation [6]. If this was not done, we could not have detected the double CBD in this case. Therefore, we assume that this asymptomatic anatomical variation may be present more commonly, but not diagnosed.

As to embryological mechanism of double CBD, Boyden proposed that the duplication of the biliary system can be present in early human embryogenesis and represents the primitive structure that regresses with normal development [1, 4] although the mechanism has been a subject of discussion.

In summary, we reported an incidentally encountered case of double CBD and discussed the novel anatomical findings of the accessory CBD from the viewpoint of embryology. Surgeons, endoscopists, and radiologists should be aware of this rare anatomical variation.

## Abbreviation

CBD: Common bile duct
